# Surgery

**Published:** 1905-09-02

**Authors:** 


### Progress in Medicine and Surgery.

### SURGERY.

**Acid Intoxication and its Surgical Significance.**—Acetone or di-acetic acid are present in minute traces in normal urine, but they occur in excess under many different pathological conditions. Kelly[1] enumerates 16 of these, which may be grouped under—Metabolic disturbances, *e.g.* diabetes mellitus, starvation, auto-intoxications; toxic conditions, *e.g.* poisoning by phosphorus, morphia, after general anæsthesia, certain septic conditions, and infectious fevers; digestive disturbances, and some injuries to the central nervous system. The great clinical importance of the condition lies in the fact that it may simulate various acute abdominal diseases, *e.g.* peritonitis or intestinal obstruction, and that the prolonged anæsthesia necessary for dealing with such conditions greatly aggravates the acid intoxication. It is of the utmost importance, therefore, that the true nature of such cases should be recognised, and that surgical interference should be avoided. A series amounting to no less than 46 in 400 consecutive surgical cases were observed in the Boston Children's Hospital. And of these six proved fatal. The first symptom usually noticed is apathy followed by vomiting, and accompanied by the sweet-smelling breath which is the most characteristic feature common to all the cases. The vomiting occurs without apparent cause soon after taking food, and is often foul with a strong acid odour. The pulse is of increased frequency and diminished tension. Fever is absent except towards the close of severe cases, when the temperature may rise to 102° or 103° F. Stupor or delirium mark the final stages, and photophobia or dimness of vision are sometimes noticed. Of the 46 reported cases 14 had peritonitis (generally caused by appendicitis) or some other acute abdominal disease, and 14 were cases of fracture.; 13 patients were 10 years old or under. Of the fatal cases one, a boy of nine, had symptoms pointing to appendicitis, was operated on, but nothing definite was found; the second, a woman of 31, had had recurrent vomiting for some months, and a posterior gastro enterostomy was performed with the idea that she had a gastric ulcer; the third, a girl of 12, had multiple infective osteomyelitis; the fourth had a fæcal fistula near the appendix of seven years' duration, no operation was performed; the fifth, a boy of 19, had persistent vomiting, with slight gastric dilatation, for which a gastro-enterostomy was done; and the sixth, a girl of 16, had extensive burns, and after these had been healing well she developed vomiting and diarrhœa, with other symptoms of acid intoxication. The post-mortem showed nothing but enlargement of the lymph glands, and Peyer's patches in the abdomen. The only methods of treatment suggested are large doses of alkalis, which seem to have but little effect, and hypodermic injections of adrenalin, which increase the blood-pressure.

**On the Prevention of Shock.**—One of the most notable recent advances in surgery has been the prevention of shock by blocking the main nerve trunks of the affected part by injections of cocaine. The possibility of this was first demonstrated by Crile experimentally. Its value has been shown practically in amputation of the forequarter and the hip. Hartwell[2] showed before the New York Surgical Society a man of 55 in whom he had amputated the arm and shoulder girdle by this method. Twenty-one years before he had fallen and struck the right shoulder; 15 years ago he had first noticed a lump on the shoulder, which had steadily increased ever since. At the time of operation it was 9 inches by 5 inches in diameter, and a mass of glands were felt in the axilla. Von Berger's operation was performed, the main vessels being first secured after a part of the clavicle had been removed. Before any of the nerve trunks were cut they were injected with a solution of cocaine, with the result that the pulse rate and tension were not affected by the operation and practically no shock was experienced. The tumour proved to be an alveolar sarcoma (endothelioma) arising from the periosteum of the clavicle. Cobb[3] relates a similar case in a young man who for only eight weeks had had a painful enlargement of the head of the humerus. A preliminary operation having proved this to be a large round-celled sarcoma, the upper limb was removed. The nerve trunks were injected with Schleich's cocaine solution before section. The operation occupied one hour and a quarter and no shock whatever was produced by it, the pulse remaining at 80 throughout and not varying at all in volume or tension. This is all the more remarkable because hitherto the mortality from this operation has been generally due to shock. The patient died nine months later from malignant disease of the pleura and lung.

[1] Annals of Surgery, Feb. 1905. [2] Ibid, Dec. 1904. [3] Ibid. Feb. 1905.

*(To be continued.)*

